# Adipokines, Biomarkers of Endothelial Activation, and Metabolic Syndrome in Patients with Ankylosing Spondylitis

**DOI:** 10.1155/2014/860651

**Published:** 2014-03-18

**Authors:** Fernanda Genre, Raquel López-Mejías, José A. Miranda-Filloy, Begoña Ubilla, Beatriz Carnero-López, Ricardo Blanco, Trinitario Pina, Carlos González-Juanatey, Javier Llorca, Miguel A. González-Gay

**Affiliations:** ^1^Epidemiology, Genetics and Atherosclerosis Research Group on Systemic Inflammatory Diseases, Rheumatology Division, IDIVAL, 39011 Santander, Spain; ^2^Rheumatology Division, Hospital Lucus Augusti, 27003 Lugo, Spain; ^3^Oncology Division, Hospital del Bierzo, 24411 Ponferrada, León, Spain; ^4^Cardiology Division, Hospital Lucus Augusti, 27003 Lugo, Spain; ^5^Computational Biology, School of Medicine, University of Cantabria, IDIVAL, and CIBER Epidemiología y Salud Pública (CIBERESP), 39011 Santander, Spain

## Abstract

Ankylosing spondylitis (AS) is a chronic inflammatory rheumatic disease associated with accelerated atherosclerosis and increased risk of cardiovascular (CV) disease. AS patients also display a high prevalence of features clustered under the name of metabolic syndrome (MeS). Anti-TNF-**α** therapy was found to be effective to treat AS patients by suppressing inflammation and also improving endothelial function. Previously, it was demonstrated that a short infusion of anti-TNF-**α** monoclonal antibodyinfliximab induced a rapid and dramatic reduction in serum insulin levels and insulin resistance along with a rapid improvement of insulin sensitivity in nondiabetic AS patients. The role of adipokines, MeS-related biomarkers and biomarkers of endothelial cell activation and inflammation seem to be relevant in different chronic inflammatory diseases. However, its implication in AS has not been fully established. Therefore, in this review we summarize the recent advances in the study of the involvement of these molecules in CV disease or MeS in AS. The assessment of adipokines and biomarkers of endothelial cell activation and MeS may be of potential relevance in the stratification of the CV risk of patients with AS.

## 1. Introduction 

Ankylosing spondylitis (AS) is a chronic inflammatory rheumatic disease, which mainly affects the axial joints, including the spine, sacroiliac joints, and entheses, but it may also involve peripheral joints [1]. Along with disease progression, inflamed joints tend to fuse (ankylosis) and there is also an ossification of the inflamed entheses, often leading to a loss of the well-known flexibility of the spine. AS is more prevalent in men than in women and usually appears around the third decade of life [1]. Moreover, extra-articular manifestations such as uveitis, psoriasis, or osteoporosis are frequently associated with this rheumatologic disease [2].

As observed in other rheumatologic diseases, such as rheumatoid arthritis (RA), AS patients disclose an increased risk of cardiovascular (CV) disease when compared to general population, being CV diseases one of the main causes of mortality in these patients [1]. Furthermore, an accelerated atherosclerotic process in these patients has also been reported [3].

AS patients also display a high prevalence of features such as obesity, dyslipidemia, hypertension, alterations in glucose metabolism, and insulin resistance (IR), which are clustered under the name of metabolic syndrome (MeS) [4]. Interestingly, individuals that suffer MeS also exhibit a dysregulation of adipokines, which are highly bioactive substances secreted by adipocytes and immune cells and that are involved not only in metabolic functions but that also play an immunomodulatory role [5, 6]. This dysregulation leads to metabolic disorders such as IR [5], an essential feature of MeS that has been associated with inflammation [7]. In addition, multiple evidences show that IR promotes endothelial dysfunction [8, 9], an early key step in the atherogenic process which appears even before the structural changes associated with this process [10].

Regarding therapeutic approaches aimed to treat AS, anti-TNF-*α* therapy was found to be effective to treat patients with this disease and other types of spondyloarthritis [11–13]. Anti-TNF-*α* agents neutralize this cytokine leading to suppression of inflammation and, consequently, to a reduction of disease activity [14]. Moreover, it was demonstrated that this biologic therapy improves endothelial function in AS patients [15].

For the purpose of this review, we took advantage of data obtained from a series of 30 nondiabetic AS patients undergoing anti-TNF-*α* therapy with the chimeric anti-TNF-*α* monoclonal antibody infliximab [16]. At the time of assessment, these patients had been treated with this biologic agent for a median of 23 months. Since IR promotes endothelial dysfunction [8, 9], while anti-TNF-*α* treatment improves endothelial function in AS patients [15], our first objective was to evaluate short-term insulin response following anti-TNF-*α* infliximab therapy. We observed that our patients experienced a rapid and dramatic reduction in serum insulin levels and IR along with rapid improvement of insulin sensitivity after a single administration of infliximab [16]. This observation had previously been described in patients with RA undergoing anti-TNF-*α* infliximab therapy [17, 18].

**Figure 1:**
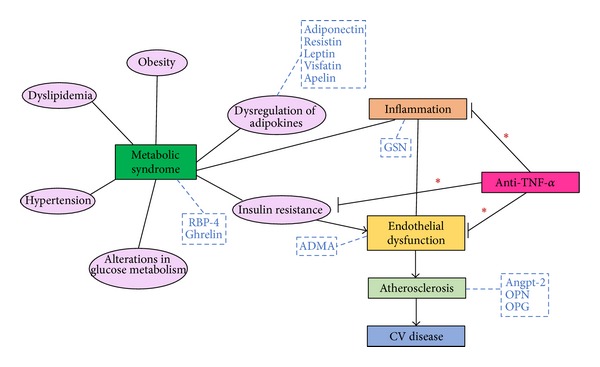
Pathophysiologic context that encompasses all the molecules reviewed in this paper. Ankylosing spondylitis patients display a high incidence of features clustered under the name of metabolic syndrome, which include obesity, dyslipidemia, hypertension, alterations in glucose metabolism, including insulin resistance, and also a dysregulation of adipokines. Moreover, all these pathologic features are associated with inflammation and lead to endothelial dysfunction and, consequently, to an enhanced risk of CV disease (mainly due to accelerated atherosclerosis) and CV death in these patients. Anti-TNF-*α* treatment not only suppresses inflammation, reducing thus ankylosing spondylitis activity, but it also improves endothelial function in these patients. The molecules that will be reviewed in this paper are included in this figure inside blue dashed boxes. *Anti-TNF-*α* improves insulin resistance and endothelial function and also reduces inflammation. ADMA: asymmetric dimethylarginine; Angpt-2: angiopoietin-2; OPG: osteoprotegerin; OPN: osteopontin; RBP-4: retinol binding protein-4.

Considering these results, we decided to further evaluate the short-term effect of anti-TNF-*α* therapy in our series of AS patients on periodical treatment with infliximab on MeS-related biomarkers, adipokines, and biomarkers of endothelial cell activation and inflammation.  Figure 1 depicts the pathophysiologic context that encompasses all the molecules reviewed in this paper. Furthermore, the main results derived from these studies on the effect of an infliximab infusion are summarized in Table 1.

In this review, recent advances in the study of the involvement of these molecules in CV disease or MeS in AS patients are discussed, along with the possible link between these biomarkers and/or adipokines and clinical characteristics of this rheumatic disease.

## 2. Metabolic Syndrome-Related Biomarkers in AS

As previously mentioned, AS patients frequently display features of MeS [4]. Therefore, the study of potential biomarkers involved in the development of such features and its association with the pathogenesis of this spondyloarthritis could give us hints for the outcome and treatment of these patients.

### 2.1. Ghrelin

Ghrelin, a peptide predominantly expressed in the stomach, is the endogenous ligand for the growth hormone secretagogue receptor (GHS-R), which regulates food intake and GH expression [19] and also acts as an anti-inflammatory molecule [20]. Previously, low levels of this peptide have been observed in obese individuals [21], a condition directly associated with hyperinsulinemia and IR. In our series we disclosed a significant correlation between ghrelin and IR and insulin sensitivity [22]. Similar results had previously been reported in individuals without rheumatic diseases [23, 24]. However, since only 10% of our patients were obese, possibly other mechanisms different from obesity may account for our findings. Furthermore, in this study we also observed a positive correlation between ghrelin and resistin, which is in accordance with their role in glucose homeostasis and the inflammatory process [22].

To our knowledge, the only previous study performed to evaluate ghrelin levels in AS patients was the one reported by Toussirot et al. [25]. However, in that study they described higher levels of ghrelin in AS patients when compared to controls. These results seem to be unexpected, since ghrelin is known to inhibit the production of inflammatory cytokines [20]. In line with this, RA patients show decreased ghrelin levels when compared to healthy controls [26]. Therefore, further studies are needed to elucidate whether the different inflammatory burden in RA and AS may account for these contradictory results.

We previously reported a significant elevation of ghrelin serum concentration upon a single infliximab infusion in RA patients with severe disease who despite receiving this anti-TNF-*α* agent had active disease with persistent elevation of laboratory markers of inflammation [27]. However, following a single infusion of infliximab, when we compared ghrelin levels found immediately before and after an infliximab infusion (the drug was administered in the fasting state, in saline solution over 120 minutes), we only disclosed a mild but not significant increase of ghrelin serum concentration in patients with AS [22]. These disparate results may be due to the absence of severe disease in our series of patients of AS at the time of assessment. It was not the case in RA patients undergoing infliximab therapy as the series of patients with RA still had active disease despite periodical treatment with this TNF-*α* inhibitor.

### 2.2. Retinol Binding Protein-4 (RBP-4)

Retinol binding protein-4 (RBP-4) is another metabolic syndrome-related biomarker, also considered a new potential cardiometabolic risk factor. This proinflammatory protein is mainly released by adipocytes but also expressed in liver and macrophages [28] and has been associated with IR in individuals with obesity, impaired glucose tolerance or type 2 diabetes mellitus, and nonobese subjects with or without family history of type 2 diabetes [29–31]. In our study, we disclosed a marginally significant correlation between RBP-4 serum levels and IR and a correlation with systolic blood pressure. Besides, when our AS patients where stratified according to sex, men showed higher levels of this protein than women [32], which was in keeping with the results previously reported by Gavi et al. [30]. More importantly, when we evaluated the effect of a single infusion of anti-TNF-*α* infliximab on RBP-4 levels, we observed a statistically significant reduction in its levels. Furthermore, this change was more evident in those patients who had a higher IR index [32].

A former study disclosed that AS patients may have lower RBP-4 serum levels than controls [33]. However, when we compared our patients with healthy controls we did not find significant differences in RBP-4 serum levels [32]. The low disease activity at the time of study in our series of AS patients may explain the absence of differences in RBP-4 serum levels when compared with controls.

## 3. Adipokines in AS

Immune system and metabolism are linked through a network of soluble mediators widely known as adipokines, which take part in both metabolic and immunomodulatory functions [6]. Although the role of adipokines seems to be relevant in many chronic inflammatory diseases, the implication in AS has not been completely elucidated.

### 3.1. Adiponectin

Adiponectin is an adipokine mainly produced by adipocytes but that can also be found in endothelial cells, skeletal muscle cells, and cardiac myocytes [6]. Circulating adiponectin levels inversely correlate with adiposity [5], which suggests that this adipokine exerts a protective function against CV disease and obesity. Adiponectin increases fatty acid oxidation and reduces the synthesis of glucose in the liver and other tissues [34]. Depending on the context, this adipokine can have pro- or anti-inflammatory functions. Unlike observations in nonrheumatic patients, RA high levels of this adipokine have been reported in the inflamed joints, since it promotes matrix degradation [35]. However, results obtained in AS are contradictory. Some groups did not find differences in serum adiponectin levels between AS patients with active disease and controls [25], while others found significantly higher levels of this adipokine in a series of AS patients under treatment with infliximab, when compared with controls [36].

In our series of AS patients we found a positive correlation between adiponectin serum levels and insulin sensitivity, suggesting that low circulating adiponectin concentrations may be associated with metabolic abnormalities that promote CV disease in AS [37]. The effect of adiponectin on insulin sensitivity is mediated in part by its ability to activate signaling pathways that lead to glucose uptake in muscle tissue and the inhibition of gluconeogenesis in the liver [38]. Furthermore, AS patients with hip involvement or synovitis and/or enthesitis in other peripheral joints had higher levels of adiponectin than those who did not have these complications [37]. These results are in keeping with the proinflammatory role described for adiponectin in the joints of RA patients [35]. Therefore, higher adiponectin levels might help to establish a subgroup of AS patients with predominant peripheral involvement.

As observed in RA patients undergoing anti-TNF-*α* infliximab therapy [39], a single infusion of this biologic agent did not lead to significant changes in the serum levels of adiponectin in patients with AS [37].

### 3.2. Resistin

Resistin is another proinflammatory adipokine mainly produced by monocytes and macrophages [40]. Proinflammatory factors such as TNF-*α* and IL-6 induce its expression [41]. Interestingly, a positive correlation between serum resistin and C-reactive protein (CRP) and erythrocyte sedimentation rate (ESR) has been observed in RA patients [42–44]. Moreover, resistin serum levels have been found increased in synovial fluid of RA patients [45]. In AS patients, likewise, higher levels of this adipokine have been observed as compared to controls [46].

In our cohort of AS patients treated with the TNF-*α* antagonist infliximab we did not observe any correlation between resistin concentration and disease activity or laboratory markers of inflammation, probably due to the low inflammatory burden as a result of prolonged treatment with anti-TNF-*α* [37]. These results are in agreement with those obtained by Kocabas et al., who did not find any correlation between resistin concentration and ESR, CRP, or BASDAI in their series of AS patients [46]. Once again, the lower inflammatory burden in our AS patients due to prolonged treatment with this biologic therapy could also explain the lack of effect observed after a single infusion of infliximab on resistin concentration [37].

### 3.3. Leptin

Leptin also belongs to the group of proinflammatory adipokines, being produced by many cell types, including adipocytes [47]. This adipokine is involved in body weight regulation, since it inhibits food intake and stimulates energy expenditure [48]. Moreover, a proatherogenic role for leptin has been described [49]. Leptin induces the production of the proinflammatory cytokines IL-6, IL-12, and TNF-*α* by monocytes and macrophages [50], while it suppresses the production of IL-4, an anti-inflammatory cytokines [51]. Leptin levels are regulated by inflammatory mediators such as TNF-*α* and IL-1 [52]. While in RA leptin has a clear proinflammatory role, showing increased levels in patients when compared with healthy controls [53], the results obtained for this adipokine in AS are contradictory. Some groups found increased leptin levels in AS patients with active disease when compared to controls [54], while others found lower circulating levels of this adipokine than in controls [25, 55].

In contrast to previous reports which found a correlation between leptin levels and BASDAI in a series of AS patients [54], in our AS cohort, we did not observe any correlation between the levels of this adipokine and clinical and laboratory parameters of disease activity and inflammation [56]. Similar results were obtained in a series of RA patients undergoing periodical anti-TNF-*α* therapy [57]. Furthermore, as previously described [58], when our series of AS patients were stratified according to sex, we disclosed higher levels of leptin in women [56].

When we analyzed the effect of TNF-*α* blockade on circulating leptin levels, we found that they were not significantly altered [56]. Similar results were obtained by other groups, either in AS patients after 6 months of infliximab treatment [36] or in RA patients undergoing 2 weeks and 6 months TNF-*α* blockade [59].

### 3.4. Visfatin

Visfatin, also known as pre-B cell colony-enhancing factor or PBEF, is a proinflammatory adipokine with ubiquitous expression [60]. Visfatin was reported to act as an insulin-mimetic adipokine [61]. This adipokine positively correlates with visceral fat [62] and has also been described as an immunomodulatory molecule [63, 64]. In fact, visfatin can induce monocytes to produce proinflammatory cytokines such as IL-1, TNF, and IL-6 [64]. Previously, increased levels of this adipokine have been observed in patients with RA [53] when compared to controls. However, to our knowledge, in addition to our study [56], the only previous study performed in AS patients was performed by Hulejová et al. [65]. In that study, which included AS patients with mild to moderate disease, no correlation was found between visfatin levels and disease activity, functional status, or acute-phase reactants [65]. Similarly, in our series of AS patients on periodical treatment with the anti-TNF-*α*-blocker infliximab, we could not find correlation between serum visfatin levels and clinical and laboratory parameters of disease activity and inflammation [56]. This was also the case for RA patients undergoing anti-TNF-*α* infliximab therapy [66].

Although we could not find association between visfatin serum levels and metabolic syndrome in RA patients with severe disease undergoing anti-TNF-*α* therapy [66], we observed a positive correlation between visfatin serum levels and IR in AS [56]. A former study performed in lean women with polycystic ovary syndrome obtained similar results [67]. However, in our series of AS patients visfatin levels did not change upon infliximab administration [56].

### 3.5. Apelin

Apelin is a quite recently new adipokine produced by diverse cell types, including adipocytes and endothelial cells [68]. This adipokine is considered a potential biomarker for CV disease risk since it stimulates nitric oxide (NO) release and, therefore, triggers arterial vasodilation [69]. Moreover, insulin directly upregulates the expression of apelin [70], making this adipokine an attractive candidate to be studied in metabolic disorders such as type-2 diabetes. Furthermore, low apelin levels have been associated with high LDL levels [71] and biomarkers of endothelial cell activation such as VCAM-1 and E-selectin correlated to apelin levels [72].

It has been postulated that apelin may have a role in the pathogenesis of the CV disease, since low levels of this adipokine have been observed in patients with ischemic heart disease [73]. However, contradictory results have been reported in patients with type 2 diabetes mellitus. In this regard, in a study fasting plasma apelin levels correlated positively with IR in patients with type 2 diabetes mellitus [74], while in another study plasma apelin levels were reduced in newly diagnosed and untreated patients with type 2 diabetes mellitus [75].

di Franco et al. measured apelin levels in early stage RA patients and found that they were lower than those observed in controls [76], suggesting a potential involvement of apelin in the pathogenesis of the rheumatic diseases. However, regarding AS, there are no previous reports performed to evaluate the levels of this adipokine in this disease. In a study of our group, apelin levels showed no association with markers of disease activity or MeS in patients with AS [77]. In line with our results, Ferraz-Amaro et al. did not find any correlation between the levels of apelin in RA patients treated with anti-TNF-*α* and BMI or IR [78].

In a further step, we analyzed the potential effect of anti-TNF-*α* infliximab treatment on apelin concentration in our series of AS patients. We found that even after the administration of a single dose of infliximab apelin serum levels were reduced, this decrease did not achieve statistical significance [77]. Similar results were previously described in RA patients after 12 months of anti-TNF-*α* treatment [78] or after 12 months of treatment with disease modifying antirheumatic drugs [76].

## 4. Biomarkers of Endothelial Cell Activation and Inflammation

In physiological conditions, NO acts as an anti-inflammatory molecule, maintaining the vascular wall in a quiescent state, also avoiding cellular proliferation. However, in the presence of inflammation or CV risk factors such as hypertension, hypercholesterolemia, or diabetes, the quiescent endothelium can switch to an activated phenotype. This leads to the secretion of proinflammatory factors such as cytokines and adipokines, to the expression of adhesion molecules for the recruitment of inflammatory cells to the vascular wall and to the generation of reactive oxygen species [79]. This pathological inflammatory condition also leads to a reduction in the release of NO into the arterial wall, either affecting its synthesis or due to oxidative inactivation of NO [80, 81], which further enhances the inflammatory status and maintains the endothelial activated phenotype [79].

Many biomarkers of endothelial cell activation and inflammation may be potentially used by clinicians to make an early diagnosis of CV disease and MeS. The implication of these biomarkers in the pathogenesis of the rheumatic diseases is also an issue of potential interest. However, as previously mentioned for adipokines, studies performed on AS patients are limited.

### 4.1. Asymmetric Dimethylarginine (ADMA)

Asymmetric dimethylarginine (ADMA) is an endogenous inhibitor of the nitric oxide synthase (NOS), causing therefore a reduction in NO production and leading to endothelial dysfunction and CV events [82]. Consequently, ADMA has been proposed as a biomarker for endothelial dysfunction and a risk factor for CV disease [82, 83]. Furthermore, increased levels of ADMA have been associated with hypertension [84], hypertriglyceridemia [85], hypercholesterolemia [86], diabetes mellitus [87], and IR [88], as well as with inflammatory diseases such as RA [76] and AS [89–91].

We found higher concentrations of ADMA in AS patients with hypertension [92]. Unexpectedly, we observed a significant negative correlation between ADMA levels and total cholesterol (TC) and LDL-cholesterol in AS [92], which apparently seems to contradict previous results that associated high ADMA levels with patients with hypercholesterolemia [86]. We think that this negative association between ADMA and TC and LDL-cholesterol might probably be the result of the long-term treatment with anti-TNF-*α* in our series of AS patients (almost 2 years), which may have led to a reduction of the inflammatory burden in these patients [14] along with complex lipidic changes [93, 94]. With respect to this, our series of patients with AS were in a state of low disease activity, with low levels of CRP, showing a BASDAI less than 3, which is indicative of a favorable disease activity state [92]. This could be the reason why we could not see statistically significant associations between CRP/disease activity markers and ADMA levels in our series of patients.

Furthermore, we did not observe any change on ADMA levels after a single infusion of anti-TNF-*α* [92]. This was in accordance with previous studies that described no effect on ADMA concentration following long-term TNF-*α* blockade [95, 96].

### 4.2. Angiopoietin-2 (Angpt-2)

Angiopoietin-2 (Angpt-2) is a proinflammatory marker of endothelial cell activation that is involved in angiogenesis and makes the endothelium responsive to inflammatory cytokines [97]. Angpt-2 levels have been found increased in recent onset RA patients with CV disease when compared with those without CV disease [97], which suggests that it could be a potential biomarker for the development of CV disease. Interestingly, a recent study of our group disclosed a correlation between age at the time of disease onset in patients with RA and Angpt-2 levels [98]. More importantly, after adjustment for sex, age at RA diagnosis,and CV risk factors, Angpt-2 levels were higher in RA patients with CV disease than in RA patients without CV complications [98]. Interestingly, in our series of AS patients undergoing infliximab therapy, we also found an association between Angpt-2 serum levels and the age at the onset of symptoms of AS, as well as a marginally significant association with disease duration [99].

It has been reported that Angpt-2 promotes the proinflammatory activation of human macrophages in RA synovial tissue and that the neutralization of this molecule in an* in vivo* model of RA decreased disease severity, inflammation, neovascularisation, and joint destruction [100]. In line with this observation, we disclosed that a single infusion of anti-TNF-*α* infliximab was associated with a dramatic reduction of Angpt-2 serum levels [99], possibly as part complex mechanisms leading to a reduction of the risk of CV events associated with this intervention in patients with chronic inflammatory diseases [101].

### 4.3. Osteopontin (OPN)

Osteopontin (OPN) is another biomarker of atherosclerosis, synthesized by osteoclasts, osteoblasts, chondrocytes, and by cells of the immune system [102, 103]. This protein has pleiotropic functions such as cellular adhesion, migration, angiogenesis, and inflammation [102, 104]. High OPN levels have been proposed to promote the development of atherosclerotic lesions [105] and atherosclerotic plaque rupture, acting as a chemotactic factor for inflammatory cells and leading thus to plaque rupture [106].

In a previous study performed by Choi et al., OPN levels were increased in AS when compared to healthy controls [102]. However, we did not observe significant differences in the levels of OPN between AS patients undergoing anti-TNF-*α* therapy and controls [107]. These different results may be due to the long-term treatment with anti-TNF-*α* therapy that our AS patients had received at the time of the study. It is possible that prolonged anti-TNF-*α* blockade may lead to reduction of the inflammatory burden and, therefore, to a reduction of OPN concentrations. In this regard, OPN levels observed in our cohort of AS patients and in the healthy matched controls were very similar to those observed in the control group reported by Choi et al. [102].

Interestingly, in our series of AS patients we found a positive correlation between serum levels of OPN and Angpt-2 [107]. Since, as described in the previous section, Angpt-2 is a marker of endothelial cell activation involved in angiogenesis, making the endothelium responsive to inflammatory cytokines [97], while OPN is also involved in the development of atherosclerotic disease, this result reinforces the idea of the potential use of OPN and Angpt-2 as biomarkers to predict CV risk in patients with AS.

As previously mentioned, a single infusion of the anti-TNF-*α* monoclonal antibody infliximab led to a significant reduction in Angpt-2 serum levels in our series of AS patients [99]. Likewise, a single infusion of infliximab also triggered a decrease in OPN serum levels in our cohort of AS patients [107]. This is in accordance with the proinflammatory role proposed for these biomarkers of endothelial cell activation and atherosclerosis and the beneficial effect against the development of CV disease mediated by the use of anti-TNF-*α* therapy.

### 4.4. Gelsolin (GSN)

Gelsolin (GSN) is an anti-inflammatory protein mainly secreted by muscle cells, that is, involved in cytoskeleton reorganization [108]. GSN acts by binding to actin filaments (and probably to other extracellular matrix components) and thus prevents the activation of downstream inflammatory pathways by these filaments [109]. Reduced GSN levels have been reported in situations of acute injury or inflammation [109–111]. In this regard, it was proposed that in inflammatory diseases such as RA, circulating GSN may be potentially locally consumed by the interaction with macromolecules such as actin, fibrin, and fibronectin at the joints or other affected organs, leading thus to a reduction of the levels of GSN [109]. In keeping with these findings we observed lower levels of GSN in AS patients undergoing anti-TNF-*α* therapy when compared to healthy controls [112]. These data support the potential function of GSN as an anti-inflammatory molecule.

As observed for other biomarkers and adipokines, a single infusion of anti-TNF-*α* infliximab did not produce any significant change on GSN serum levels in our AS patients [112]. As pointed out before, a possible explanation for this steady level of GSN might be the low disease activity of our patients, since they had been receiving infliximab for a long period of time.

### 4.5. Osteoprotegerin (OPG)

Osteoprotegerin (OPG) is a member of the TNF receptor superfamily, that is, implicated both in osteoporosis and in the atherosclerotic process. OPG acts as a decoy receptor for the receptor activator of nuclear factor-*κ*B ligand (RANKL), inhibiting binding of RANKL to its receptor, RANK [113, 114]. OPG also acts as a soluble neutralizing receptor of TNF-related apoptosis-inducing ligand (TRAIL), an anti-inflammatory molecule with antiatherosclerotic properties [115–117]. Furthermore, it has been reported that OPG can upregulate the production of endothelial adhesion molecules [118].

OPG has previously been associated with increased risk of atherosclerotic disease in the general population [119]. Interestingly, a recent study of our group on patients with RA undergoing infliximab therapy disclosed that OPG concentrations were associated with biomarkers of endothelial activation (intercellular adhesion molecule-1), carotid intima-media wall thickness, and carotid plaques [120]. In keeping with these results, in our series of AS patients undergoing infliximab therapy we have disclosed an independent correlation with ADMA, another biomarker of endothelial cell activation [121]. This further supports the role of OPG as a valuable CV disease risk biomarker in chronic inflammatory rheumatic diseases such as AS.

Although a significant reduction of OPG levels upon infusion of this biologic agent was recently reported in long-standing RA patients with severe disease undergoing anti-TNF-*α* therapy [120], a single administration of anti-TNF-*α* infliximab did not lead to any significant reduction of OPG levels in our series of AS patients [121]. The low disease activity and low inflammatory burden observed at the time of the study in our series of AS patients could probably explain the lack of significant reduction of OPG levels following administration of anti-TNF-*α*.

## 5. Therapeutic Potential Applications of These Biomarkers and Adipokines

Constantly, a progressively increasing list of new potential biomarkers and adipokines comes out, being subject to evaluation for their involvement in CV disease or MeS. However, as wisely suggested by other authors, these molecules exert such complex physiological effects that their use as potential therapeutic molecules must be carefully planned to obtain the adequate result and to avoid unknown side effects [5, 40].

Another point of potential interest is the possible use of these molecules as biomarkers of CV disease and predictors of increased risk for CV events or MeS in patients with diseases like AS that are associated with increased risk of CV death. However, up to now the routine use of these biomarkers in the daily clinical practice is still far from being well established. It is applicable not only to individuals with chronic inflammatory rheumatic diseases like AS or RA but also to the general population.

## 6. Conclusions

The assessment of adipokines and biomarkers of endothelial cell activation and MeS may be of potential interest for the improvement of the stratification of the CV risk of patients with AS. However, further studies are still needed to fully elucidate the clinical implication of these molecules in the mechanisms leading to accelerated atherosclerosis in AS and the benefits of the assessment of these molecules in the daily clinical practice.

## Figures and Tables

**Table 1:** Effect of an infusion of the anti-TNF-*α* monoclonal antibody infliximab on MeS-related biomarkers, biomarkers of endothelial cell activation and inflammation, and adipokines in a series of AS patients undergoing periodic treatment with this drug.

Target	Biologic effect
MeS-related biomarkers	Reduction in serum insulin levels and IR Improvement of insulin sensitivity Reduction of RBP-4 serum levels No significant change on ghrelin serum levels

Biomarkers of endothelial cell activation and inflammation	Reduction of Angpt-2 serum levels Reduction of OPN serum levels No significant change on ADMA and GSN serum levels No significant reduction on OPG plasma levels

Adipokines	No significant change in the levels of the different adipokines (adiponectin, resistin, leptin, visfatin, and apelin)
